# Progress in Surgery

**Published:** 1898-06-11

**Authors:** 


### PROGRESS IN SURGERY.

### SURGERY OF THE CHEST.

**Pleural Effusions.**—Dr. Samuel West[1] has recently related two cases of serous pleural effusion which he treated by incision and drainage with successful results. The first case was that of a lady, aged thirty-one, who had had an effusion for more than twelve months. Repeated aspiration having failed to cure this the chest was opened and drained as for empyema, and ultimately the patient recovered perfectly without chest deformity. The second case was that of a man, aged thirty-nine, who had had the right side of the chest full of fluid for twelve months. There was a strong family history of consumption, and the man himself was probably phthisical. Tapping of the chest, repeated three times, having done no permanent good, an incision was made into the pleura and a drainage tube inserted. No portion of rib was excised. The lung completely expanded in a week, except where the tube lay. The discharge became purulent. The patient ultimately returned to work in good health, but still wearing the drainage tube, which it was found impossible to remove. Dr. West alluded to the satisfactory way in which the lung had expanded in these cases though so long compressed—eighteen and fifteen months respectively. He protested against the notion that compression of the lung prevented the development of tubercle, or that tubercle tended to develop rapidly when the compression was removed. Mr. Spencer, commenting on the above cases, recommended routine removal of a rib when operating to drain the pleura. It is obviously unsatisfactory to let a patient go about with a drainage tube in the chest, for amylaceous disease is likely to develop ultimately.

Dr. B. F. Curtis,[2] of New York, has published a paper on "The Treatment of Chronic Empyema." It has become fashionable to attribute the failure to cure an empyema to the fault of the surgeon, to his delay or his imperfect methods in operating. Curtis points out how latently pus may form in the chest. It it not very rare to find a labouring man still working with his chest full of fluid. No blame attaches to the surgeon in such cases for the delay in operating. Again, the writer points out that all wounds do not heal, and suppuration does not always cease with incision and with the maximum care in the use of antiseptic methods. Tuberculosis and probably lack of resistance in the patient have a good deal to do with the failures in the treatment of these cases. The formation of granulation tissue in the pleura is small in degree. The great thickening of the pleura is a consequence of the deposition of fibrin which becomes organised. The cavity has to be obliterated by contact of the parietes and the lung through the recession of the former and expansion of the latter. The great thickening of the pleura resists both of these movements, and also the ribs may come into contact and prevent efficient parietal shrinking.

The names of Estlander, Schede, Létievant, and lastly of Delorme are associated with the operations in vogue for the assistance of those processes by which the cavity of chronic empyema is obliterated. Estlander removes ribs only over the cavity. Schede, in addition, removes the thickened parietal pleura. Delormè has successfully removed the visceral pleura from the surface of the lung, and so allowed it to re-expand. To do this he divided the ribs along the line of the incision, and temporarily turned them back in a flap. Delorme states that the hæmorrhage is not excessive in this operation. For the prevention of development of the chronic sequelæ of empyema, which the above operations are required to cure, early and efficient drainage must be secured, and this is best done by making a free opening low down and far back in the chest, with removal of a portion of rib. Curtis mentions a case in which a well-known surgeon, operating on the above lines, wounded the diaphragm, the central part of which was depressed, and passed the drainage tube through the uppermost part of the peritoneal cavity and the diaphragm into the pleura. The patient died of peritonitis. To avoid such an accident the operation should be done deliberately, each structure being divided after identification. Remarking upon the management of the drainage tube, Curtis says it should be long enough, as the cavity contracts to a flat space between the chest wall and the lung, to reach within half an inch of the apex of the cavity. There is no question that a reaccumulation of pus will sometimes follow the use of too short a tube, and still more often this evil follows early removal of the tube. Chronic empyemata are total, partial, or in the form of persistent sinuses, and may be closed or open. If the pleura is tuberculous cure may be impossible. It must be remembered, however, that an empyema in a tuberculous subject may not be due to tubercular pleuritis, and may therefore be more amenable to treatment. Curtis lays stress upon the danger of operating on a closed empyema which communicates with a bronchus, and mentions a case where death quickly followed an operation in such a case, although only light ether anæsthesia was used, and the patient sat upright. Coughing followed the entry of air into the pleura, and thence into the lung, and pus was forced by the cough into the sound lung. There was the interesting pathological condition of complete calcification of the pleura, both visceral and parietal, in the above case, the plate being a sixteenth of an inch thick.

When should the operations of Estlander or Schede be performed? In ordinary acute cases which have been drained Curtis does not operate unless the condition has been stationary a month or six weeks, but if the empyema had existed a long time before being drained he would not wait more than a fortnight. It is certain that better results follow the operations in which the thickened pleura, the periosteum of the ribs, and the intercostal muscles are removed, than those in which bone only is removed. It is becoming common, therefore, even in dealing with the smaller cavities, to remove all these structures. There are two ways of dealing with the skin and muscle flaps. The ordinary way is to pack the cavity and bring the flaps over the packing. Schede's method, necessary in the graver cases, is to fold the flaps over the ends of the divided ribs and apply them directly to the surface of the lung and line the cavity as far as possible. It is important during convalescence to assist lung expansion by the use of a spirometer.

Dr. W. B. Hopkins,[3] before the College of Physicians of Philadelphia, described an operation for empyema in which the patient became very much depressed after the escape of three quarts of pus, respiration being noticeably feeble. In order to overcome these symptoms the cavity was syphoned full of warm sterile water which was retained for about four minutes until the patient had somewhat improved, and then allowed slowly to escape. Dr. Hopkins urged also the use of a valvular drainage tube in these cases, establishing a negative pressure in the pleura, and thus aiding expansion of the lung in an early stage of the disease before the lung became bound down.

[1] Brit. Med. Jour., Feb., 1898. [2] Med. Rec., Mar., 1898. [3] Annals of Surgery, Feb , 1898.

### CEREBRO-SPINAL SURGERY.

**Meningocele and Spina Bifida.**—Within recent years the treatment of congenital protrusions of the meninges of the brain and spinal cord has undergone a marked change. Ligature of the neck of the sac after exposure by a cutting operation has been slowly but certainly replacing the use of iodine and other irritant injections. The current literature of the last few months illustrates a variety of conditions in which surgical intervention has been of benefit, and seems to indicate that a carefully-planned and skilfully-performed operation is attended with at least as little immediate risk to life, and with somewhat better prospects of ultimate recovery, than the injection methods of Morton and others.

In a recent paper by Curtis[1] a series of four cases treated by operation is recorded. We do not think the first of these is calculated to encourage operative measures. A baby 48 hours old, "sluggish and idiotic, and paying no attention to anything," suffered from a large hydrencephalocœle in the occipital region, in addition to being microcephalic. The sac, consisting of dura mater, was exposed by cutting two lateral flaps; the pedicle was tied in two portions after transfixion, and the tumour cut away. Death followed in 24 hours. The tumour, under the microscope, proved to be composed of degenerated nervous tissue, with a large proportion of fibrous tissue. Of three cases of spina bifida, one dorsal and one lumbo-sacral recovered after operation, while a lumbo-sacral case, complicated with hydrocephalus, proved fatal.

In commenting on his cases the writer says that the risks in operations for spina bifida in young children are hæmorrhage, loss of cerebro-spinal fluid, shock, and sepsis. The co-existence of hydrocephalus and paralysis of lower extremities, bladder, and rectum contra-indicate operation for the spina bifida, unless rupture is imminent. Rapidity in operating; avoidance of hæmorrhage by opening the sac in the middle, and so only dividing terminal vessels; securely closing the neck of the sac, by silk preferably, and only after returning any nerve trunks which may have been extruded; and, of course, asepsis, are the main factors towards success. The author is not convinced of the practicability of closing the defect in the neural arch by periosteal flaps borrowed from adjacent bone.

As examples of successful operations in patients of more advanced age may be mentioned two cases reported and figured by Whitehead.[2] One was a boy age 11, with a large lumbo-sacral meningocœle, which had gradually increased from a swelling two inches in diameter to one the size of a child's head. He was slightly hydrocephalic, but with no mental defect. He had talipes calcaneo-valgus, complete paralysis of lower limbs, incontinence of urine and fæces, as well as trophic sores on the legs. As the skin over the swelling threatened to give way operation was advised. About a pint of cerebro-spinal fluid escaped on opening the sac, the neck of which was closed by fine silk sutures, the excess being cut away. A free discharge of cerebro-spinal fluid took place for some weeks, eventually becoming purulent. Although the operation wound finally closed, leaving a sound strong cicatrix, little or no materal benefit to symptoms accrued. In the same paper illustrations are given of a similar case successfully treated by the author in 1883. Clutton[3] reports a very satisfactory operation for spinal bifida in a woman aged 26. The tumour measured about 15 inches in diameter, and projected from the middle of the sacrum. It was translucent, and did not appear to contain any solid element. On excising the whole sac with a portion of thinned skin over it, a fine opening was found communicating with the spinal canal, and from this clear fluid escaped. The opening was closed with catgut, and the skin with silkworm gut. Eleven days after operation a large quantity of cerebro-spinal fluid burst through the cicatrix, and continued to ooze for three weeks. No other untoward symptoms followed. The same surgeon operated with complete success on an identical case some years ago.

Two very creditable results of operation for occipital meningocœle are reported by Spanton.[4] They occurred in his practice within a few weeks of each other. In both, after evacuating clear fluid, the neck was secured by a Staffordshire knot, and no subsequent leakage took place. In neither case did any brain disturbance follow the operation.

Clutton[3] reports a very interesting case of congenital sacro-coccygeal tumour successfully excised from a child three years old. The tumour had its origin in post-anal gut, had grown *pari passu* with the child, and when operated upon hung down from the buttocks to about half-way to the knees. It was of the nature of a glioma and was partly cystic (dermoid).

**Potts' Disease and its Results.**—The diagnosis of Potts' disease in its earliest stages is often a matter of great difficulty. Grognot[5] discusses the early painful manifestations, and points out that these are not only very variable both in degree and site, but that they tend to change their position, a point on which he lays considerable stress. Bilateral pain, or pain shifting from one side to the same part on the opposite side, for example, in the line of the sciatic or intercostal nerves, is strongly suggestive of spinal mischief. Deep-seated pains, simulating muscular rheumatism, or lumbago, and arthralgic pains, especially in the knee, are often met with. All these pseudo-neuralgic pains are commoner in adults than in children. Paralytic symptoms, of course, give a clearer indication as to the nature of the mischief.

In discussing the treatment of spinal abscess Duplay[6] expresses the opinion that operation should be avoided longer in children than in adolescents and adults. In children the injection of a 1 in 10 ethereal solution of iodoform sometimes gives good results; less frequently in adults. The best operative treatment is by curetting, the application of zinc chloride, and drainage.

Traap[7] reports a case in which compression paralysis from an intra-dural collection of tubercular débris was relieved by laminectomy.

**Non-Tubercular Affections of the Spine.**—An interesting discussion took place recently in the New York Academy of Medicine on a paper by Myers[8] on "Non-Tuberculous Inflammation of the Spine." (1) Syphilitic: These are especially difficult to diagnose. The cervical spine is the most common situation, probably because of the proximity of the throat and pharynx. Townsend believes this condition to be rare, about three or four in a thousand. The coexistence of a dactylitis is not much aid in diagnosis. (2) Rheumatic: Rheumatoid arthritis affects the cervical spine least. It is never accompanied by suppuration. Ransom has very seldom seen rheumatoid arthritis confined to the spine. There is no tenderness on vertical pressure or on walking. Townsend thinks that in rheumatic disease the kyphosis is more rounded and less distinct than in tubercle. (3) Malignant: Myers considers this a rare condition. It is usually secondary and may be carcinomatous or sarcomatous. Kyphosis is exceptional, but the spinal nerves are frequently involved in the new growths. Gibney considers the intensity of the pain and its localisation in certain regions an important aid in diagnosis. It often follows cancer of the breast in women. An example of this was quoted by Curtis in a woman 35 years of age, whose breast was removed a year before the disease appeared in the mid-dorsal region. Very marked signs of pressure on the cord and intense pain served to localise the growth, and laminectomy confirmed the diagnosis, but failed to give relief. Dowd emphasised the frequency with which malignant disease of the spine follows breast cancer. In five out of twenty-nine of his own cases it occurred. (4) Gonorrhœal disease of the spine is exceedingly rare. (5) Typhoid: An inflammatory condition of the fibrous structures of the spine has been observed to follow on typhoid fever. (6) Infectious inflammations of the spine following exanthematous diseases usually attack the cervical vertebræ, possibly due to direct spread from the throat. (7) Traumatism frequently induces inflammation of the spine. Lloyd thinks that tearing of the ligamentum subflavum, with hæmorrhage and subsequent inflammation, often simulates Potts' disease. More severe lesions can usually be correctly diagnosed. Williams[9] reports an interesting case of traumatic hæmato myelia with necropsy. (8) Hydatids: Lloyd refers to a case which began with kyphosis, followed by the development of a distinct tumour, which on removal proved to be a hydatid tumour. Wood[10]

operated with success for a similar condition in the lower cervical region in a boy aged 10. The mother cyst and 16 daughter cysts were removed. Cerebral meningitis carried off the patient 18 days after the operation. (9) The possibility of scurvy, rickets, or aneurism, giving rise to symptoms very like those of Potts' disease, must always be kept in mind.

[1] Post Graduate, March, 1898. [2] Brit. Med. Jour., Mar. 12, 1898. [3] Annals of Surgery, March, 1898. [4] Brit. Med. Jour., Oct., 1897. [5] Journal de Médicine, 1897. [6] Sem. Méd., Dec , 1897. [7] Muncher Med. Wochen, No. 27, 1897. [8] Med. Rec., Mar. 5, 1898. [9] Lancet, Aug., 1897. [10] Inter-colonial Med. Jour. of Australasia, 1897.

